# Gastrin Attenuates Renal Ischemia/Reperfusion Injury by a PI3K/Akt/Bad-Mediated Anti-apoptosis Signaling

**DOI:** 10.3389/fphar.2020.540479

**Published:** 2020-11-06

**Authors:** Chao Liu, Ken Chen, Huaixiang Wang, Ye Zhang, Xudong Duan, Yuanzheng Xue, Hongye He, Yu Huang, Zhi Chen, Hongmei Ren, Hongyong Wang, Chunyu Zeng

**Affiliations:** ^1^ Department of Cardiology, Daping Hospital, Army Medical University, Chongqing, China; ^2^ Chongqing Institute of Cardiology & Chongqing Key Laboratory of Hypertension Research, Chongqing, China; ^3^ Department of Lishilu Outpatient, General Hospital of the PLA Rocket Force, Beijing, China; ^4^ Cardiovascular Research Center of Chongqing College, Department of Cardiology of Chongqing General Hospital, University of Chinese Academy of Sciences, Chongqing, China; ^5^ State Key Laboratory of Trauma, Burns and Combined Injury, Daping Hospital, Army Medical University, Chongqing, China

**Keywords:** gastrin, CCKBR, ischemia-reperfusion injury, acute kidney injury, apoptosis, Akt

## Abstract

Ischemic/reperfusion (I/R) injury is the primary cause of acute kidney injury (AKI). Gastrin, a gastrointestinal hormone, is involved in the regulation of kidney function of sodium excretion. However, whether gastrin has an effect on kidney I/R injury is unknown. Here we show that cholecystokinin B receptor (CCKBR), the gastrin receptor, was significantly up-regulated in I/R-injured mouse kidneys. While pre-administration of gastrin ameliorated I/R-induced renal pathological damage, as reflected by the levels of serum creatinine and blood urea nitrogen, hematoxylin and eosin staining and periodic acid-Schiff staining. The protective effect could be ascribed to the reduced apoptosis for gastrin reduced tubular cell apoptosis both *in vivo* and *in vitro*. *In vitro* studies also showed gastrin preserved the viability of hypoxia/reoxygenation (H/R)-treated human kidney 2 (HK-2) cells and reduced the lactate dehydrogenase release, which were blocked by CI-988, a specific CCKBR antagonist. Mechanistically, the PI3K/Akt/Bad pathway participates in the pathological process, because gastrin treatment increased phosphorylation of PI3K, Akt and Bad. While in the presence of wortmannin (1 μM), a PI3K inhibitor, the gastrin-induced phosphorylation of Akt after H/R treatment was blocked. Additionally, wortmannin and Akt inhibitor VIII blocked the protective effect of gastrin on viability of HK-2 cells subjected to H/R treatment. These studies reveals that gastrin attenuates kidney I/R injury via a PI3K/Akt/Bad-mediated anti-apoptosis signaling. Thus, gastrin can be considered as a promising drug candidate to prevent AKI.

## Introduction

Acute kidney injury (AKI) is a commonly encountered syndrome worldwide, leading to decreased kidney function, and associated with higher mortality, morbidity, and elevated risks of development of chronic kidney disease (Zuk and Bonventre, 2016; Hoste et al., 2018). Ischemia/reperfusion (I/R) injury, usually caused by many medical conditions such as shock and organ transplantation, is one of the leading causes of AKI. Despite great progress has been made in understanding of the causes and the mechanisms of AKI, it remains a major unmet medical need, and continues to be a global public health concern impacting about 13.3 million patients per year (Mehta et al., 2015; Zuk and Bonventre, 2016). Thus, there is an urgent need to find novel therapeutic options to prevent or treat AKI effectively.

The gastrointestinal (GI) tract, the organ first exposed to components of food, excretes numerous hormones and humoral factors. Those hormones and humoral factors work via their individual receptors, which have been found to be expressed both inside and outside GI tract, including kidneys (Dufresne et al., 2006; Furness et al., 2013). Emerging evidence proves the existence of gastro-renal axis (Jose et al., 2016a), GI tract–derived hormones and peptides could regulate the autocrine function of renal hormones, affecting renal function such as sodium excretion (Michell et al., 2008; Yang et al., 2017). Numerous GI hormones, like gastrin and cholecystokinin, are secreted during a meal. The level of circulating gastrin in bloodstream is markedly increased after a meal, about 10- to 20-fold more than cholecystokinin (Rehfeld et al., 2007; Yang et al., 2018). Gastrin, a peptide hormone secreted by gastrin cells (G cells) in the stomach and duodenum, primarily stimulates parietal cells of the stomach to secrete gastric acid. More importantly, gastrin has previously been reported to have a protective effect against myocardial ischemia/reperfusion injury in rat hearts (Yang et al., 2018). Taking into account the evidence of the existence of the gastro-renal axis, we hypothesize that gastrin may attenuate kidney I/R injury. Thus, the aim of the study was to investigate the effect of gastrin on kidney I/R injury and explore the underlying mechanisms.

## Materials and Methods

### Animals

Adult male C57/BL6 mice at the age of 10–12 weeks (weighting 23–26 g) were obtained from the Laboratory Animal Center of Daping Hospital (Chongqing, China). All procedures were in accordance with institutional regulations and with the approval of the Experimental Animals Committee of Daping Hospital.

### Establishment of Renal Ischemic/Reperfusion Injury Model

The mouse model of renal I/R injury was induced according to the protocol described previously (Hesketh et al., 2014; Yakulov et al., 2018). Briefly, mice were anesthetized via intraperitoneal injection of sodium pentobarbital (60 mg/kg) and placed on a heating pad to maintain body temperature at 37°C during surgery. After clean of the surgical area, a midline laparotomy incision was performed and the right kidney was exposed. Then the right ureter, right renal artery and vein were all ligated before right nephrectomy. The left kidney was used for ischemia and reperfusion, the left renal artery was clamped by an artery clip for 40 min. Gastrin (20 μg/kg, Tocris, Bristol, United Kingdom) was administrated to the left kidney before ischemia in the gastrin group (Chen et al., 2013; Liu Z. et al., 2016; Yang et al., 2018), while the natural saline was used instead of gastrin in the control group. Besides, CI-988 (1 mg/kg, Tocris, Bristol, United Kingdom) was administrated before gastrin in the CI-988 + Gastrin group (Hinks et al., 1996; Chen et al., 2013; Yang et al., 2018). Then incisions were sutured and the mice were allowed to recover with free access to food and water. The sham group underwent the identical procedures except the left renal artery clamp. Twenty-four hours after renal reperfusion, mice were re-anesthetized after which a laparotomy and nephrectomy were performed. Blood samples were collected and kidneys were harvested and kept for further analysis.

### Renal Function

The renal function of the mice was assayed by measuring serum creatinine (SCr) and blood urea nitrogen (BUN). Blood samples were centrifuged at 600 × g for 10 min for the separation of serum. Then serum samples were analyzed by creatinine assay kit and BUN assay kit (Nanjing Jiancheng Bioengineering Institute, Nanjing, China) by enzymatic colorimetric methods according to the manufacturer’s instructions (Chen et al., 2015).

## Renal Histopathology, Periodic Acid-Schiff Staining and Immunohistochemistry Staining

Kidney samples were fixed in 4% paraformaldehyde and then dehydrated in increasing concentrations of ethanol. Thereafter, samples were cleared in xylene and embedded in paraffin. The samples were cut into 4 μm thick sections followed by a staining with hematoxylin and eosin (H&E). Each sample section was examined by a pathologist in a blind manner. Morphological changes to the tubules were scored to assess the degree of renal damage as described previously (Wang et al., 2013), these changes included tubular epithelial cell swelling, vacuolization, cast formation and desquamation. Pathological scoring ranged from 0 to 5 point based on the injury area of involvement. The scale is as follows: 0, normal; 1, histological changes involving <10% of injury area; 2, similar changes involving >10% but <25% of injury area; 3, similar changes involving >25% but <50% of injury area; 4, similar changes involving >50% but <75% of injury area; 5, similar changes involving >75% of injury area.

The Periodic Acid-Schiff (PAS) staining was performed using a PAS staining kit (Solarbio, Beijing, China) according to the manufacturer’s instruction (Zhao et al., 2017; Yu et al., 2018). In brief, sections were incubated with 0.5% periodic acid solution for 10 min, then, stained with Schiff’s reagent for 15 min, followed by counterstaining with hematoxylin solution for 2 min. Finally, sections in each group were recorded under a microscope.

The immunohistochemistry staining was carried out using a rabbit anti-CCKBR antibody (1:100, OriGene, Rockville, MD, United States), reactions were detected using horseradish peroxidase-conjugated goat anti-rabbit IgG (Zsbio, Beijing, China), and the color was developed using 3,3-diaminobenzidine tetrahydrochloride (DAB, Solarbio, Beijing, China) and stopped by rinsing in deionized water followed by microscopy.

## Cell Culture and Hypoxia/Reoxygenation Treatment

Human kidney 2 (HK-2) cells (ATCC, Manassas, VA, United States) were cultured at 37°C in DMEM/F-12 (HyClone, South Logan, UT, United States) with 1% v/v penicillin/streptomycin (Invitrogen Life Technologies, Karlsruhe, Germany) and 10% v/v fetal bovine serum (Invitrogen Life Technologies, Karlsruhe, Germany). For hypoxia/reoxygenation (H/R) treatment, cells were exposed to 24 h of hypoxia (5% CO_2_, 1% O_2_, and 94% N_2_) followed by 3 h of reoxygenation (Kwon et al., 2006; Yu et al., 2013).

### Western Blot Analysis

Western Blot (WB) analysis were performed following the protocol described in our previous study (Liu et al., 2020). Proteins were extracted from kidney tissues or HK-2 cells with ice-cold Tissue Extraction Reagent (Thermo Scientific, Waltham, MA, United States) contained with protease inhibitor cocktail (Roche, Indianapolis, IN) and phosphatase inhibitor cocktail (PhosSTOP™, Sigma-Aldrich, St. Louis, MO, United States). The homogenate was sonicated and kept on ice for 1 h. The lysate was centrifuged at 12,000 × g for 30 min. The supernatants were boiled in the loading buffer (Solarbio, Beijing, China) for 5 min at 95°C and samples were stored at −20°C before use. Protein samples (50 μg) were separated by SDS-PAGE with 10% or 15% polyacrylamide gel and electrotransferred to nitrocellulose membranes. After the non-specific binding sites were blocked in TBS (Tris-buffered saline) containing 5% non-fat dry milk for 1 h, the membranes were incubated at 4°C overnight with primary antibodies including anti-CCKBR (1:1000, OriGene, Rockville, MD, United States) anti-KIM-1 (1:500, Novus Biologicals, Littleton, CO, United States), anti-PI3K p85α (1:500, Beyotime, Jiangsu, China), anti-p-PI3K p85 (Tyr458)/p55 (Tyr199) (1:1000, Cell Signaling Technology, Danvers, MA, United States), anti-Akt (1:1000, Proteintech, Wuhan, China), anti-p-Akt (Ser473) (1:1000, Cell Signaling Technology, Danvers, MA, United States), anti-Bad (Beyotime, Jiangsu, China) and anti-p-Bad (Ser112) (Beyotime, Jiangsu, China). Anti-GAPDH (1:5000, Proteintech, Wuhan, China) was used as internal control. The membranes were then washed and incubated with the IRDye 680 labeled goat anti-rabbit or IRDye 800 labeled goat anti-mouse secondary antibody (1:15,000, Li-Cor Biosciences, Lincoln, NE, United States) at room temperature for 1 h. Membranes were washed three times in TBST, and the bounds were detected by the Odyssey Infrared Imaging System (Li-Cor Biosciences, Lincoln, NE, United States). The images were analyzed using the Odyssey Application Software to obtain integrated intensities.

## Real-Time Quantitative Polymerase Chain Reaction

CCKBR mRNA levels were quantified by Quantitative Polymerase Chain Reaction (qPCR) with the following specific primers: CCKBR, forward (5′- CAA​CAC​GTG​GCG​TGC​CTT​C-3′) and reverse (5′- GGA​ATC​GGC​GGT​GCA​TGA​AA-3′); β-actin, forward (5′-TCA​CTG​TCC​ACC​TTC​CAG​CAG​A-3′) and reverse (5′-AGG​GTG​TAA​AAC​GCA​GCT​CAG​TAA-3′).

Total RNA was extracted using RNAiso Plus reagent (TaKaRa, Tokyo, Japan) and cDNA synthesis was performed using the PrimeScript™ RT Master Mix (TaKaRa, Tokyo, Japan) according to the manufacturer’s instructions. For qPCR, the mRNA levels of CCKBR and β-actin were measured using the TB Green™ Premix Ex Taq™ (TaKaRa, Tokyo, Japan) and the Thermal Cycler Dice™ Real-Time System (Bio-rad, Hercules, CA, United States). The following PCR condition was applied: 95°C for 3 min, 40 cycles at 95°C for 10 s and 62°C for 30 s followed by 62°C 10 s. And the mRNA relative expression levels were evaluated using the 2^−ΔΔCt^ method and normalized to β-actin, the qPCR products were also analyzed by agarose gel electrophoresis.

### TUNEL Assay

TUNEL assays were performed with the DeadEnd™ Fluorometric TUNEL System (Promega, Medison, WI, United States) according to the manufacturer’s instructions. Briefly, slides were immersed in 4% paraformaldehyde for 25 min at 4°C, followed by permeabilization with 0.2% Triton^®^ X-100 in phosphate buffer saline (PBS) for 5 min. Slides were equilibrated for 5 min in manufacturer's buffer before undergoing TdT reactions for 60 min at 37°C. Reactions were stopped with 2×SSC, washed three times with PBS and mounted. To visualize all nuclei, slides were incubated with 4′, 6-diamino-2-phenyl-indole (DAPI). Finally, apoptotic cells was detected by fluorescence microscopy. The number of TUNEL-positive cells was divided by the total number of cells in each image to obtain the percentage of TUNEL-positive cells (Liu et al., 2019).

### Cell Viability Assay

Cell viability was assayed using CCK-8 (Cell Counting Kit-8, Beyotime, Jiangsu, China) according to manufacturer’s protocol. Briefly, HK-2 cells were cultured in a 96-well plate and treated with gastrin (10^−12^, 10^−11^, 10^−10^ and 10^−9^ M) or without gastrin, and PBS or CI-988 was used as control. After normoxia or H/R treatment, 10 μL CCK-8 solution/well was added and cells were incubated for another 30 min at 37°C. The amount of formazan dye generated by cellular dehydrogenase activity was measured by absorbance at 450 nm with a microplate reader.

### Lactic Dehydrogenase Assay

Lactic Dehydrogenase (LDH) is a stable cytoplasmic enzyme, which could be rapidly released into the cell culture medium upon damage of the plasma membrane, and is taken as a cell injury marker (Kumar et al., 2018). HK-2 cells were cultured in a 24-well plate in the presence of gastrin (10^−9^ M) with or without CI-988 (10^−5^ M) and then subjected to H/R treatment. PBS was used as control. The amount of LDH released from the cells was measured with LDH Assay Kit (Beyotime, Haimen, China) according to the manufacturer’s suggestion (Dong et al., 2013; Yan et al., 2015).

### Detection of the Caspase-3 Activation

Renal tissue or HK-2 cell proteins were extracted according to the protocol described in the WB analysis, and the protein concentrations were assayed using a BCA protein assay kit (Solarbio, Beijing, China). An equal quantity of total protein (10 µg) from each sample was used to measure caspase-3 activity using caspase-3 activity kits (Beyotime, Jiangsu, China). Activity of caspase-3 was measured using substrate peptides acetyl-DEVD-p-nitroanilide (Ac-DEVD-pNA) and the release of pNA was qualified by determining the absorbance with a spectrometer plate reader at 405 nm.

### Reactive Oxygen Species Measurement

For detection of the superoxide anion, HK-2 cells in each group were incubated with 5 μM dihydroethidium (DHE, Beyotime, Jiangsu, China) at 37°C for 30 min. Then cells were fixed with 4% paraformaldehyde in PBS for 15 min at room temperature. After rinsed in PBS for three times, the cells were analyzed by fluorescence microscopy.

## Detection of Malondialdehyde and Superoxide Dismutase

Levels of Detection of Malondialdehyde (MDA) and Superoxide Dismutase (SOD) in HK-2 cells were detected by the colorimetric method using commercially available kits purchased from the Jiancheng Bioengineering Institute (Nanjing, China). All procedures were performed according to the manufacturers’ instruction.

### Statistical Analysis

Data were presented as mean ± standard deviation (SD) values. Statistical significance was determined by ANOVA test followed by Sidak’s multiple comparisons test or Tukey’s multiple comparisons test for multi-group (>2) comparison and Student’s t-test was used for two-group comparison (GraphPad Prism 6.0, Graph Pad Software Inc., San Diego, CA, United States). Differences were considered statistically significant when *p* < 0.05.

## Results

### Increased Expression of CCKBR in the Ischemic/Reperfusion-Injured Mouse Kidneys

**FIGURE 1:**
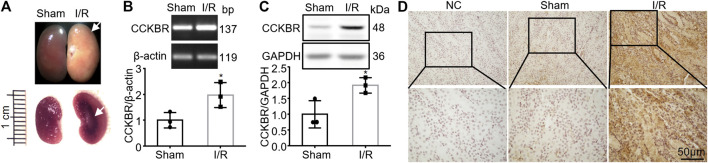
Increased expression of CCKBR in the I/R-injured mouse kidneys. **(A)** Representative pictures showing pathological changes at the surface and cortico-medullary border of the I/R-injured kidneys (scale bar, 1 cm). **(B)** The mRNA level of CCKBR was significantly increased in the kidneys after I/R injury, compared with the sham kidneys (*n* = 3, *p < 0.05 vs. Sham). **(C)** A remarkable increase of CCKBR proteins in the kidneys after I/R injury (*n* = 3, *p < 0.05 vs. Sham). **(D)** Representative images of IHC staining indicating CCKBR was expressed in tubular cells and it was up-regulated after renal I/R injury (NC: negative control; scale bar, 50 μm; magnification of ×200 in the upper panel and ×400 in the lower panel).

To investigate whether gastrin plays a role in renal I/R injury, we firstly detected if the expression of CCKBR was changed in kidneys after I/R injury. Mouse model of renal I/R injury was successfully established, and the injured kidneys showed pathological changes at the surface and cortico-medullary border compared with the sham kidneys (Figure 1A). The qPCR result showed the mRNA level of CCKBR was significantly increased in the kidneys after I/R injury, compared with the sham kidneys (Figure 1B). Moreover, the WB analysis demonstrated a remarkable increase of CCKBR proteins in the kidneys after I/R injury (Figure 1C). Additionally, IHC staining showed that CCKBR was expressed in tubular cells and its expression was increased after I/R injury (Figure 1D).

### Gastrin Improves Renal Function and Alleviates Pathological Damage After Renal Ischemic/Reperfusion Injury

**FIGURE 2:**
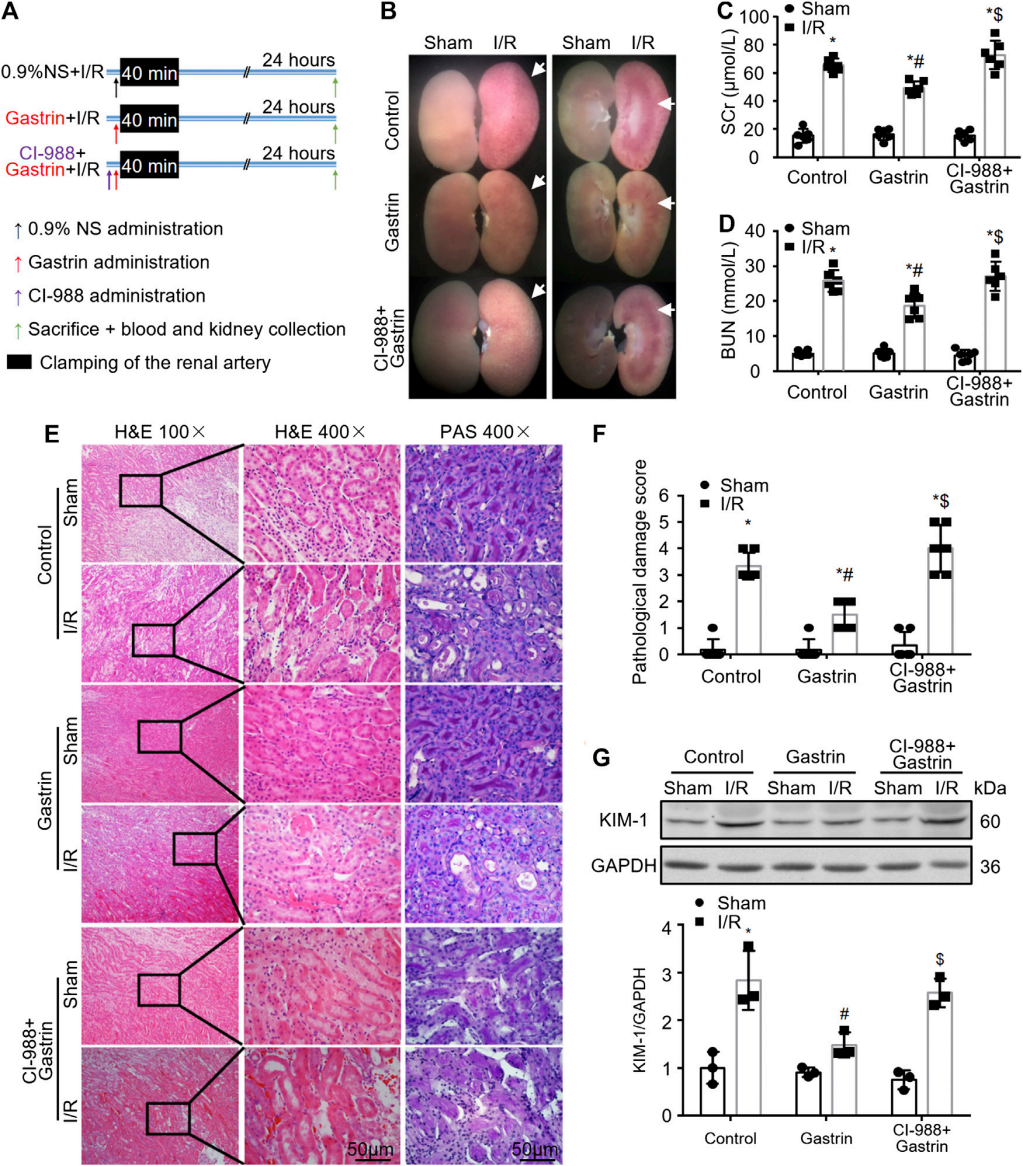
Gastrin improves renal function and alleviates pathological damage after renal I/R injury. **(A)** Schematic illustration of the protocol used for I/R-injured mouse kidneys (NS: normal saline). **(B)** Representative pictures of mouse kidney specimens revealing that gastrin pre-treatment alleviates I/R-induced damage, while CI-988 blocked the protective effect. **(C,D)** Gastrin pre-treatment mitigated I/R-induced elevation of SCr and BUN, which was inhibited by the addition of CI-988 (*n* = 6, *p < 0.05 vs. Control-Sham; #*p* < 0.05 vs. Control-I/R; $*p* < 0.05 vs. Gastrin-I/R). **(E)** Gastrin alleviated renal pathological damage, revealed by the representative images of H&E staining and PAS staining, while CI-988 inhibited the beneficial effect (scale bar, 50 μm). **(F)** The semi-quantitative score of renal pathological damage (*n* = 6, *p < 0.05 vs. Control-Sham; #*p* < 0.05 vs. Control-I/R; $*p* < 0.05 vs. Gastrin-I/R). **(G)** The expression of KIM-1, a marker for tubule injury, was less elevated in I/R-injured kidneys pre-treated with gastrin, while the effect of gastrin was abolished in the presence of CI-988 (*n* = 3, *p < 0.05 vs. Control-Sham; #*p* < 0.05 vs. Control-I/R; $*p* < 0.05 vs. Gastrin-I/R).

To further explore the effect of gastrin on renal I/R injury, gastrin (20 μg/kg) was administrated via intraperitoneal during the abdominal surgery, and the protocol was illustrated by the schematic graph (Figure 2A). We found that gastrin pre-treatment relieved the kidney damage while the beneficial effect was blocked by CI-988, as reflected by representative pictures of mouse kidney specimens (Figure 2B), the Scr levels (Figure 2C) and BUN levels (Figure 2D). Meanwhile, the H&E staining and PAS staining revealed that gastrin alleviated renal pathological damage induced by I/R, while CI-988 abolished the protective effect (Figures 2E,F). Additionally, the WB analysis showed that the expression of KIM-1, a marker for tubule injury (Han et al., 2002), was less elevated in I/R-injured kidneys pre-treated with gastrin (Figure 2G).

### Gastrin Attenuates Ischemic/Reperfusion-Induced Cell Apoptosis in Mouse Kidneys

**FIGURE 3:**
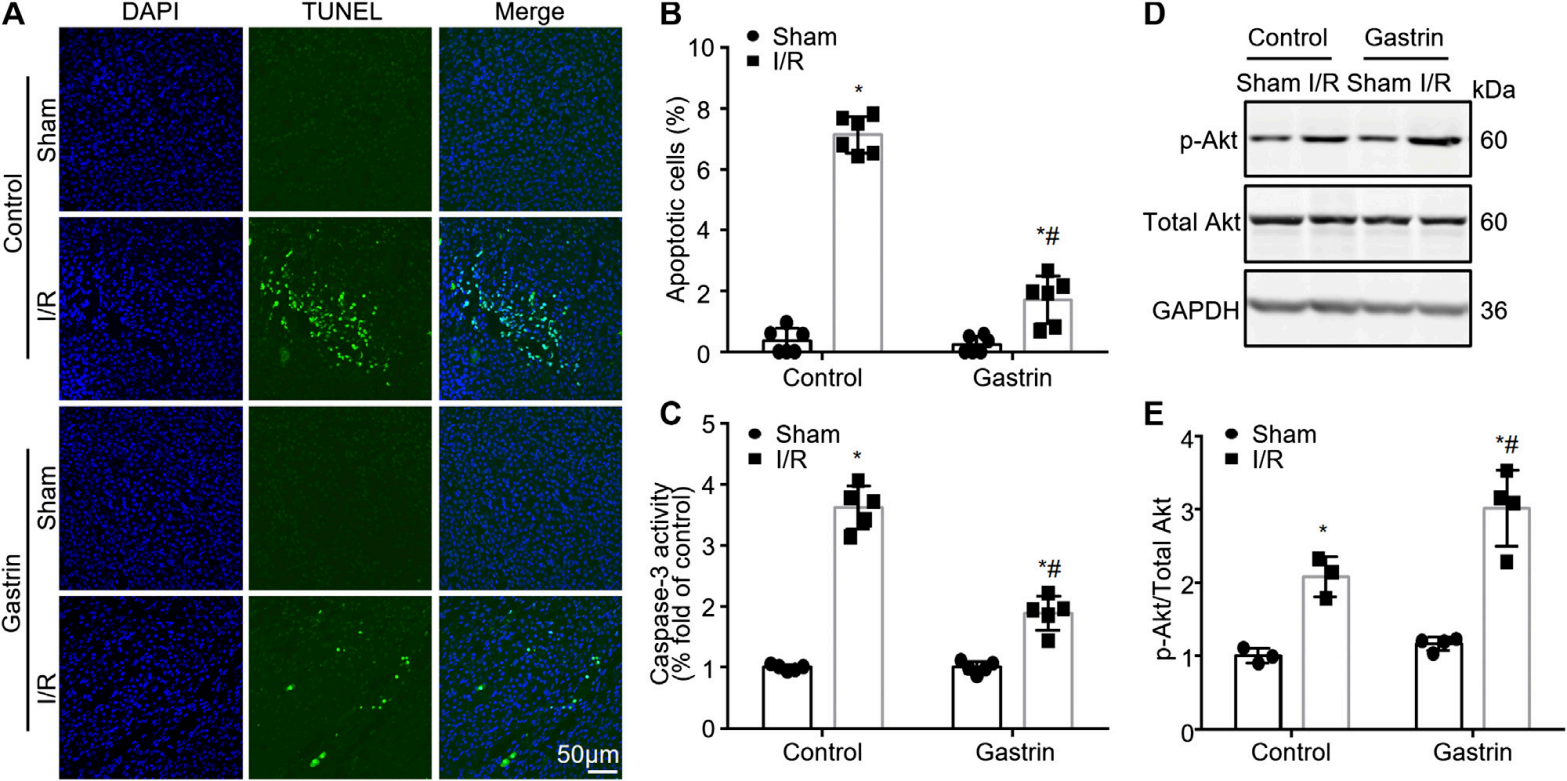
Gastrin attenuates I/R-induced cell apoptosis in mouse kidneys. **(A,B)** TUNEL staining and statistical results showing gastrin pre-treatment reduced cell apoptosis induced by I/R injury (*n* = 6, *p < 0.05 vs. Control-Sham; #*p* < 0.05 vs. Control-I/R; scale bar, 50 μm). **(C)** Gastrin protected against the I/R-induced increase in caspase-3 activity, reflected by caspase-3 activity assay (*n* = 5, *p < 0.05 vs. Control-Sham; #*p* < 0.05 vs. Control-I/R). **(D,E)** Representative images and the statistic results showing that the phosphorylation of Akt was elevated after I/R injury and gastrin pre-treatment further increased the phosphorylation of Akt (p-: phosphorylated, *n* = 6 in Control and 8 in Gastrin, *p < 0.05 vs. Control-Sham; #*p* < 0.05 vs. Control-I/R).

We next explored the effect of gastrin on I/R-induced cell apoptosis in kidneys. TUNEL assay was performed and the results showed that gastrin pre-treatment reduced cell apoptosis induced by I/R injury (Figures 3A,B). Moreover, caspase-3 activity was determined by the DVDE-pNA colorimetric assay and the results indicated gastrin protected against the I/R-induced increase in caspase-3 activity (Figure 3C). Akt (also known as protein kinase B) has been identified as a serine/threonine-specific protein kinase that plays a key role in multiple cellular processes including apoptosis (Franke et al., 2003), thus we further tested if the phosphorylation of Akt was changed after gastrin administration. The WB result showed phosphorylation of Akt was elevated after I/R injury, and gastrin pre-treatment further increased the phosphorylation of Akt (Figures 3D,E), suggesting an enhanced Akt activity after gastrin administration.

### Gastrin Preserves the Viability of Hypoxia/Reoxygenation-Treated HK-2 Cells

**FIGURE 4:**
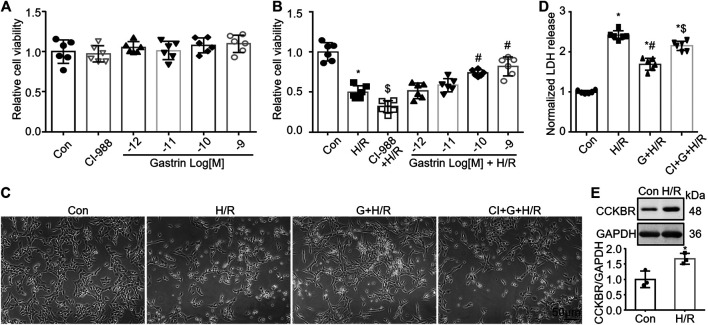
Gastrin preserves the viability of H/R-treated HK-2 cells. **(A)** No obvious effect of gastrin on cell viability of HK-2 cells under normoxic condition, indicated by CCK-8 assay (Con: Control; *n* = 6). **(B)** Gastrin preserved the viability of H/R-treated HK-2 cells in a dose-dependent manner, reflected by CCK-8 assay (H/R: hypoxia/reoxygenation; *n* = 6, *p < 0.05 vs. Control; $*p* < 0.05 vs. H/R; #*p* < 0.05 vs. H/R). **(C)** Representative images showing gastrin (10^−9^ M) reduced the H/R-induced cell death, while CI-988 (10^−5^ M) blocked the protective effect of gastrin (G: Gastrin; CI: CI-988; scale bar, 50 μm). **(D)** Gastrin reduced the LDH release from HK-2 cells after H/R treatment, while CI-988 (10^−5^ M) abolished the protective effect of gastrin (10^−9^ M) (*n* = 6, *p < 0.05 vs. Control; #*p* < 0.05 vs. H/R; $ *p* < 0.05 vs. Gastrin + H/R). **(E)** CCKBR was significantly up-regulated in HK-2 cells after H/R treatment (*n* = 3, *p < 0.05 vs. Control).

Since gastrin pre-administration alleviated I/R-induced kidney *in vivo*, we next investigated the effect of gastrin on HK-2 cells, a proximal tubular cell line derived from human normal kidney (Ryan et al., 1994), *in vitro*. No obvious effect of gastrin on cell viability of HK-2 cells under normoxia was found, as indicated by CCK-8 assay (Figure 4A), while gastrin preserved the viability of H/R-treated HK-2 cells in a dose-dependent manner (Figure 4B). In addition, representative images indicated that gastrin (10^−9^ M) reduced the H/R-induced cell death, while CI-988 (10^−5^ M) blocked the protective effect of gastrin (Figure 4C). It also reflected that gastrin reduced the LDH release from HK-2 cells after H/R treatment, but abolished by CI-988 (10^−5^ M) (Figure 4D). More importantly, in agreement with the *in vivo* experiments, the expression of CCKBR was also dramatically up-regulated in HK-2 cells after H/R treatment (Figure 4E).

### Gastrin Reduces HK-2 Cell Apoptosis and Reactive Oxygen Species Production Induced by Hypoxia/Reoxygenation Treatment

**FIGURE 5:**
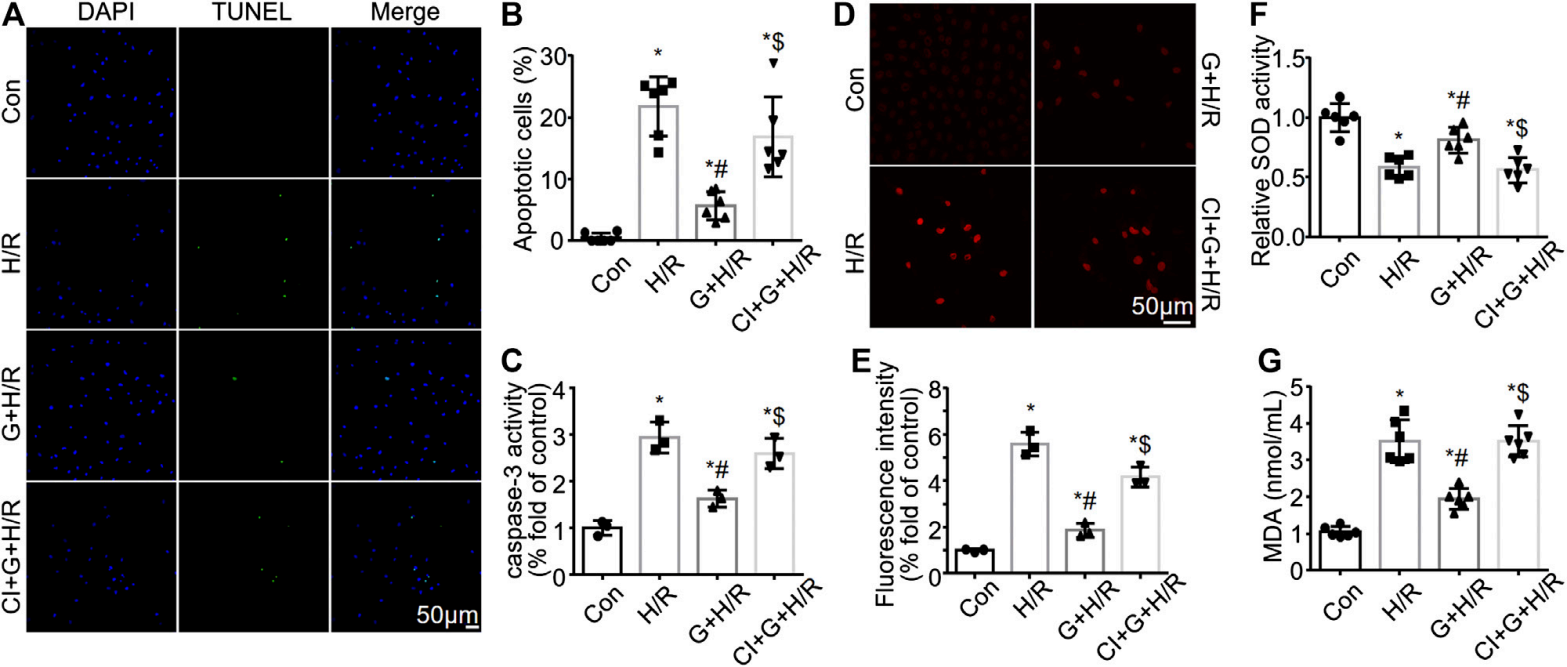
Gastrin reduces HK-2 cell apoptosis and ROS production induced by H/R treatment. **(A,B)** Representative pictures and the statistical results of TUNEL assay illustrating that gastrin (10^−9^ M) reduced HK-2 cell apoptosis induced by H/R treatment, while CI-988 (10^−5^ M) abolished the anti-apoptotic effect of gastrin (Con: Control; H/R: hypoxia/reoxygenation; G: Gastrin; CI: CI-988; *n* = 6, *p < 0.05 vs. Control; #*p* < 0.05 vs. H/R; $ *p* < 0.05 vs. Gastrin + H/R; scale bar, 50 μm). **(C)** Gastrin (10^−9^ M) alleviated the H/R-induced increase in caspase-3 activity in HK-2 cells, while CI-988 (10^−5^ M) reduced the effect of gastrin, indicated by caspase-3 activity assay (*n* = 3, *p < 0.05 vs. Control; #*p* < 0.05 vs. H/R; $ *p* < 0.05 vs. Gastrin + H/R). **(D,E)** Representative images and statistical results of DHE staining indicating that gastrin (10^−9^ M) reduced the H/R-induced ROS production in HK-2 cells, while CI-988 (10^−5^ M) abolished the effect of gastrin (*n* = 3, *p < 0.05 vs. Control; #*p* < 0.05 vs. H/R; $ *p* < 0.05 vs. Gastrin + H/R; scale bar, 50 μm). **(F)** Gastrin (10^−9^ M) preserved the H/R-caused SOD activity reduction in HK-2 cells, while CI-988 (10^−5^ M) abolished the effect of gastrin (*n* = 6, *p < 0.05 vs. Control; #*p* < 0.05 vs. H/R; $ *p* < 0.05 vs. Gastrin + H/R). **(G)** Gastrin (10^−9^ M) ameliorated the H/R-induced increasing of MDA level in HK-2 cells, while CI-988 (10^−5^ M) blocked the effect of gastrin (*n* = 6, *p < 0.05 vs. Control; #*p* < 0.05 vs. H/R; $ *p* < 0.05 vs. Gastrin + H/R).

While gastrin preserved the viability of H/R-treated HK-2 cells, we further investigated the effect of gastrin on HK-2 cell apoptosis after H/R treatment. TUNEL assay illustrated that gastrin (10^−9^ M) reduced HK-2 cell apoptosis induced by H/R treatment, while CI-988 (10^−5^ M) abolished the anti-apoptotic effect of gastrin (Figures 5A,B). Meanwhile, gastrin (10^−9^ M) also alleviated the H/R-induced increase in caspase-3 activity in HK-2 cells which was blocked by CI-988 (10^−5^ M), which was reflected by the DVDE-pNA colorimetric assay (Figure 5C). Reactive Oxygen Species (ROS) has been regarded to be tightly associated with I/R injury and cell apoptosis (Chien et al., 2001), thus we also tested if gastrin would have an effect on ROS production. DHE staining indicated that gastrin (10^−9^ M) reduced the H/R-induced ROS production in HK-2 cells, while CI-988 (10^−5^ M) abolished the effect of gastrin (Figures 5D,E). Additionally, gastrin (10^−9^ M) preserved the H/R-caused SOD activity decreasing in HK-2 cells, while CI-988 (10^−5^ M) abolished the effect of gastrin (Figure 5F). Gastrin (10^−9^ M) also ameliorated the H/R-induced increasing of MDA level in HK-2 cells, while CI-988 (10^−5^ M) blocked the effect of gastrin (Figure 5G).

### PI3K/Akt/Bad Pathway Is Involved in the Gastrin-Mediated Protective Effect Against Hypoxia/Reoxygenation

**FIGURE 6:**
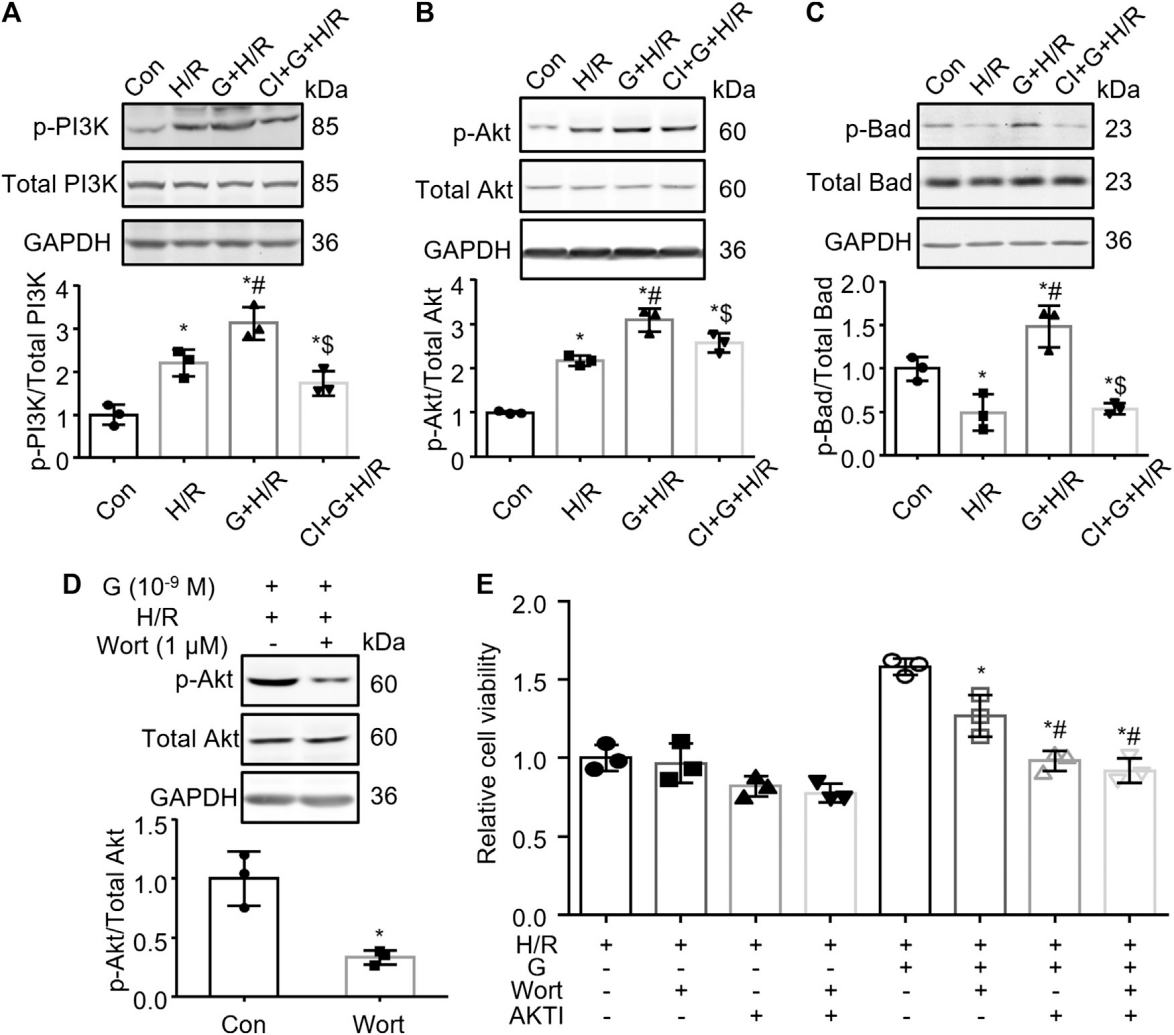
PI3K/Akt/Bad pathway is involved in the gastrin-mediated protective effect against H/R injury. **(A)** Gastrin (10^−9^ M) increased H/R-induced phosphorylation of PI3K, while CI-988 (10^−5^ M) blocked the effect of gastrin (p-: phosphorylated; Con: Control; H/R: hypoxia/reoxygenation; G: Gastrin; CI: CI-988; *n* = 3, *p < 0.05 vs. Control; #*p* < 0.05 vs. H/R; $*p* < 0.05 vs. Gastrin + H/R). **(B)** Gastrin (10^−9^ M) increased H/R-induced phosphorylation of Akt, while CI-988 (10^−5^ M) abolished the effect of gastrin (*n* = 3, *p < 0.05 vs. Control; #*p* < 0.05 vs. H/R; $*p* < 0.05 vs. Gastrin + H/R). **(C)** Gastrin (10^−9^ M) increased phosphorylation of Bad after H/R treatment, while CI-988 (10^−5^ M) reduced the effect of gastrin (*n* = 3, *p < 0.05 vs. Control; #*p* < 0.05 vs. H/R; $*p* < 0.05 vs. Gastrin + H/R). **(D)** Wortmannin (1 μM) blocked the gastrin-induced phosphorylation of Akt after H/R treatment (Wort: Wortmannin; *n* = 3, *p < 0.05 vs. Control). **(E)** Wortmannin (1 μM) and Akt inhibitor VIII (10 μM) reduced the protective effect of gastrin on viability of HK-2 cells subjected to H/R treatment, determined by CCK-8 assay (AKTI: AKT inhibitor VIII; *n* = 3, *p < 0.05 vs. the fifth group; #*p* < 0.05 vs. the sixth group).

Previous studies have shown that gastrin can activate phosphatidylinositol 3 kinase (PI3K) (Todisco et al., 2001; Subramaniam et al., 2008), while the PI3K/Akt/Bad pathway has been considered to play a vital role in the regulation of cell apoptosis. Thus we further explored whether the PI3K/Akt/Bad pathway was involved in the gastrin-mediated protective effect against H/R. The WB analysis showed that gastrin (10^−9^ M) further increased the H/R-induced phosphorylation of PI3K and Akt, which was blocked by the co-administration of CI-988 (10^−5^ M) (Figures 6A,B). Meanwhile, gastrin (10^−9^ M) also increased the phosphorylation of Bad after H/R treatment, while CI-988 (10^−5^ M) co-administration reduced the effect of gastrin (Figure 6C). More importantly, wortmannin (1 μM, a PI3K inhibitor) blocked the gastrin-induced phosphorylation of Akt after H/R treatment (Figure 6D), suggesting that gastrin-induced phosphorylation of Akt was via PI3K. Besides, the CCK-8 assay indicated that Wortmannin (1 μM) and Akt inhibitor VIII (10 μM) significantly reduced the protective effect of gastrin on viability of HK-2 cells subjected to H/R treatment (Figure 6E).

## Discussion

Our study provides evidence that gastrin attenuates I/R-induced AKI in mouse models. I/R injury increases the expression of CCKBR in the kidneys while gastrin pre-administration ameliorates the I/R-induced renal pathological damage and function deterioration. *In vitro* studies show that gastrin preserves the viability of H/R-treated HK-2 cells and reduces H/R-induced HK-2 cell apoptosis. Mechanistically, gastrin activates the PI3K/Akt/Bad signaling pathway via CCKBR to exert the anti-apoptotic effect.

In the past few years, several studies have suggested that gastrointestinal hormones are involved in the regulation of renal function (Von et al., 2000; Banday and Lokhandwala, 2013; Jose, 2016; Jose et al., 2016a; Jose et al., 2016b). Gastrin has been reported to interact with dopamine receptor to modulate the renal sodium excretion and thus participates in the regulation of blood pressure (Chen et al., 2013; Jiang et al., 2016). Moreover, gastrin has been reported to decrease the sodium-hydrogen exchanger three and Na^+^, K^+^-ATPase activity in RPT cells (Liang and Jose, 2012; Gildea et al., 2015; Liu T. et al., 2016). However, the role of gastrin in renal I/R injury has never been investigated. In our study, we demonstrated that the pre-treatment of gastrin reduced the I/R-induced renal injury in mice for the first time. Our results provided the evidence to support the novel concept of the gastro-renal axis. CCKBR, also named the gastrin receptor, is primarily expressed in the central nervous system and gastrointestinal tract (Nishimura et al., 2015), but CCKBR was also reported to be expressed in kidneys, especially in tubular cells (Weerth and Schrenck, 1998). Our study proved that CCKBR was expressed at both mRNA and protein levels in mouse kidneys. More interestingly, the expression of CCKBR was significantly up-regulated after the renal I/R injury, suggesting a possible role of gastrin in the renal I/R injury. Our results also showed that pre-administration of gastrin reduced the I/R-induced elevation of SCr and BUN in mice, and it also ameliorated the renal pathological damage as reflected by the H&E staining and PAS staining.

Proximal tubule epithelial cells are highly susceptible to apoptosis (Havasi and Borkan, 2011). Numerous studies have provided the evidence that tubular cell apoptosis plays an important role in the pathophysiology of AKI (Bonegio and Lieberthal, 2002; Saikumar and Venkatachalam, 2003; Chen et al., 2008; Havasi and Borkan, 2011). We also found that I/R induced a significant increase in tubular cell apoptosis in mouse kidneys. To simulate the process of renal I/R *in vivo*, we used the HK-2 cells subjected to H/R *in vitro* as did in previous studies (Kwon et al., 2006; Yu et al., 2013). The result showed that gastrin preserved the viability of H/R-treated HK-2 cells in a dose-dependent manner, as reflected by the CCK-8 assay. Besides, the LDH release, another method to access the cell death and cytotoxicity, was also measured, which showed that gastrin reduced the LDH release from HK-2 cells after H/R treatment while it was abolished by CI-988 (10^−5^ M). These data implied a beneficial effect of gastrin on H/R-treated HK-2 cells *in vitro.* We also found that H/R remarkably increased HK-2 cell apoptosis *in vitro*, and thus we next explored the role of gastrin in cell apoptosis. Previous studies showed a controversial result about the effect of gastrin on cell apoptosis. Some literatures proved that gastrin increased cell apoptosis (Kanno et al., 2001; Cui et al., 2006; Sebens Müerköster et al., 2008), while other reports indicated that gastrin inhibited cell apoptosis (Kun et al., 2017; Yang et al., 2018), indicating the underlying complex regulatory mechanisms. Our study proved that gastrin alleviated the I/R-induced tubular cell apoptosis in mouse kidneys and also reduced the H/R-induced HK-2 cell apoptosis *in vitro*. ROS has been regarded to be tightly associated with H/R injury and cell apoptosis, thus we also tested if gastrin would have an effect on ROS production *in vitro*. The result of DHE staining and levels of MDA and SOD indicated a beneficial effect of gastrin on alleviating the H/R-induced oxidative stress in HK-2 cells.

We further investigated the underlying mechanisms and found that the protective effect of gastrin was associated with the PI3K/Akt pathway, which is a classical intracellular signal transduction pathway that promotes metabolism, proliferation, cell survival, growth and angiogenesis in response to extracellular signals. It was reported that the cardioprotective effect of gastrin is abolished by inhibitors of Akt (Yang et al., 2018). Our present study showed that gastrin preserved the viability and reduced the apoptosis of H/R-treated HK-2 cells, which was abolished by CI-988 (a CCKBR antagonist), indicating the protective effect of gastrin was via CCKBR. More importantly, gastrin pretreatment induced the phosphorylation of PI3K, Akt and Bad, and wortmannin (a specific PI3K inhibitor) blocked the gastrin-induced phosphorylation of Akt, suggesting a role of PI3K/Akt/Bad pathway in the gastrin-mediated protective effect. Interestingly, H/R alone decreased the phosphorylation of Bad, but increased the phosphorylation of PI3K and Akt, as also reported previously (Chen et al., 2007; Gong and Wang, 2018). The elevation of p-PI3K and p-Akt might be compensation mechanisms. Furthermore, the CCK-8 assay proved that in the presence of wortmannin and Akt inhibitor VIII, the protective effect of gastrin was markedly inhibited. Phosphorylated Akt could further phosphorylate the downstream Bad (Datta et al., 1997; Franke and Cantley, 1997), which could then inhibit the mitochondrial injury and the initiation of intrinsic apoptosis pathway (Datta et al., 2002). In line with those conclusions, our study showed the protective effect of gastrin was associated with decreased ROS level (as reflected by the DHE, SOD and MDA levels) and reduced caspase-3 activity. Taking together, these results proved the gastrin reduce apoptosis via CCKBR to activate PI3K/Akt/Bad pathway. However, we have not investigate other pathways downstream of the CCKBR since there is a complex regulatory network, and more attention may be paid to it in future studies.

We also realized the limitations in our present study. For example, the gastrin was administrated before ischemia, which would limit its application in the “real” situation in clinical practice and thus restrict its translational potential. Therefore, the therapeutic effect of gastrin also needs to be done in the future.

## Conclusion

**FIGURE 7:**
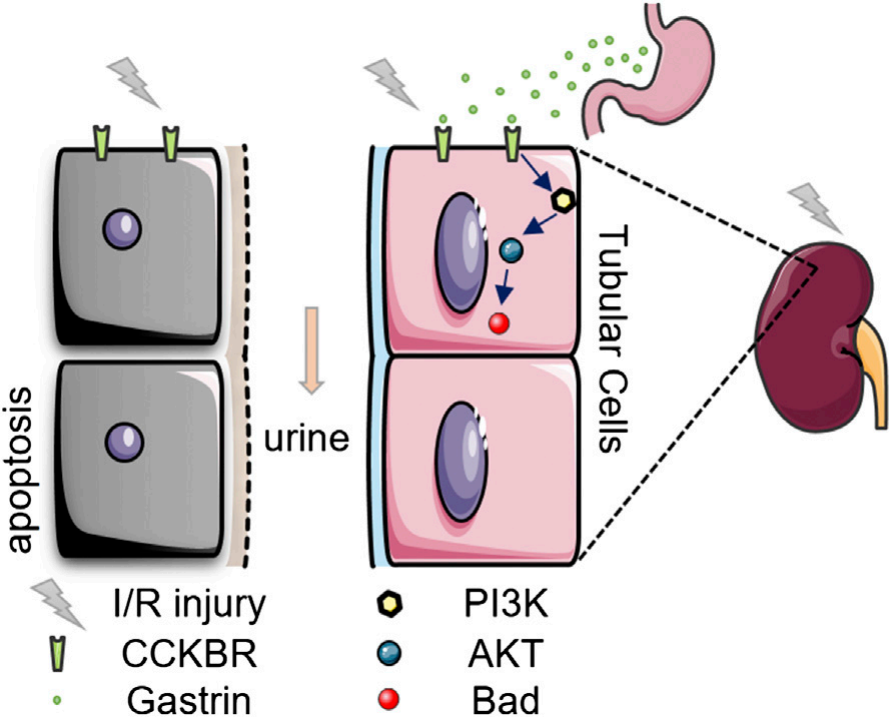
A schematic representation of the protective effect of gastrin on renal I/R injury.

Our present study suggests that pre-administration of gastrin could attenuates kidney I/R injury via CCKBR to activate PI3K/Akt/Bad-mediated anti-apoptosis signaling (Figure 7), and gastrin might be a potential effective means to prevent AKI in clinic.

## Data Availability Statement

The raw data supporting the conclusions of this manuscript will be made available by the authors, without undue reservation, to any qualified researcher.

## Author Contributions

CZ supervised the whole experiments. CL, KC and HXW designed this study. CL, YZ, and XD performed the experiments. YX, HH, YH, ZC, HR and HYW provided help during experiments.

## Funding

These studies were supported, in part, by grants from the National Key R&D Program of China (2018YFC1312700), National Natural Science Foundation of China (31730043, 31430043), Program of Innovative Research Team by National Natural Science Foundation (81721001), Program for Changjiang Scholars and Innovative Research Team in University (IRT1216), Clinical medical research talent training program of TMMU (2018XLC10I2), Chongqing Technology Innovation and Application Demonstration Project (cstc2018jscx-mszdX0024).

## Conflict of Interest

The authors declare that the research was conducted in the absence of any commercial or financial relationships that could be construed as a potential conflict of interest.
